# Camel Milk Shelf Life Optimization: Physicochemical Deterioration, Bioactive Preservation, and Non-Thermal Technologies: A Narrative Review

**DOI:** 10.3390/foods15152614

**Published:** 2026-07-26

**Authors:** Nour A. Elsahoryi, Omar A. Alhaj, Haitham Jahrami

**Affiliations:** 1 Department of Nutrition, Faculty of Pharmacy and Medical Sciences, University of Petra, Amman 11196, Jordan; omar.alhaj@uop.edu.jo; 2 Government Hospitals, Manama 329, Bahrain; hjahrami@hospitals.gov.bh; 3 Department of Psychiatry, College of Medicine and Medical Sciences, Arabian Gulf University, Manama 329, Bahrain

**Keywords:** camel milk, shelf life, physicochemical deterioration, bioactive proteins, lactoferrin, immunoglobulins, non-thermal processing, active packaging

## Abstract

Camel milk (CM), which is produced by both *Camelus dromedarius* and *Camelus bactrianus*, is gaining increasing popularity as a value-added functional dairy product, but the science of managing its shelf life has progressed more slowly than its commercial expansion. Unlike bovine milk, CM lacks β-lactoglobulin (β-LG), has a unique casein (CN) micelle structure with a high β-CN-to-αs1-CN ratio, and low κ-CN protein content, and contains an unusually rich bioactive protein fraction including lactoferrin (LF), immunoglobulin G (IgG), lysozyme (LZ), and peptidoglycan recognition protein (PGRP). These compositional characteristics imbue CM with both significant inherent antimicrobial benefits and place it at risk of processing weaknesses that do not have direct analogs in bovine dairy science. The review discusses three key areas of CM shelf life science that have not been sufficiently addressed in the published literature. The first relates to physicochemical mechanisms of deterioration, including lipid oxidation of polyunsaturated fatty acids (PUFA), destabilization of CN micelles in the structural absence of β-LG, and Maillard browning during thermal treatment and storage of powder. The second addresses the fate of bioactive proteins, with the majority being lactoferrin (LF), immunoglobulin G (Igs), and heavy chain-only antibodies (HCAbs), under both thermal and non-thermal processing conditions. The third looks at new non-thermal preservation methods, such as high-pressure processing (HPP), pulsed electric field (PEF), ultrasonication, ultraviolet irradiation, cold plasma, and electromagnetic (EM) field treatment, and functional packaging innovations, which contribute to longer shelf life. Of the non-thermal options examined, HPP at 200–400 MPa today provides the most mechanistically proven evidence for camel-specific microbial deactivation with satisfactory quality preservation. The latest primary CM research also indicates that processing with EMs in the 850 mT range can potentially be as effective as conventional pasteurization in microbial control and may be more effective than pasteurization in retaining desirable bioactive markers, although this result needs to be independently replicated before more responsible conclusions can be drawn. The review ends with four priority research gaps: standardized kinetic shelf life modeling frameworks, systematic characterization of bioactive protein stability under commercial processing conditions, life cycle assessment of preservation chains across the camel dairy supply continuum, and the urgent validation of camel-specific pasteurization adequacy indicators to replace bovine alkaline phosphatase, which remains active in CM after conventional high-temperature short-time (HTST) pasteurization.

## 1. Introduction

**Figure 1:**
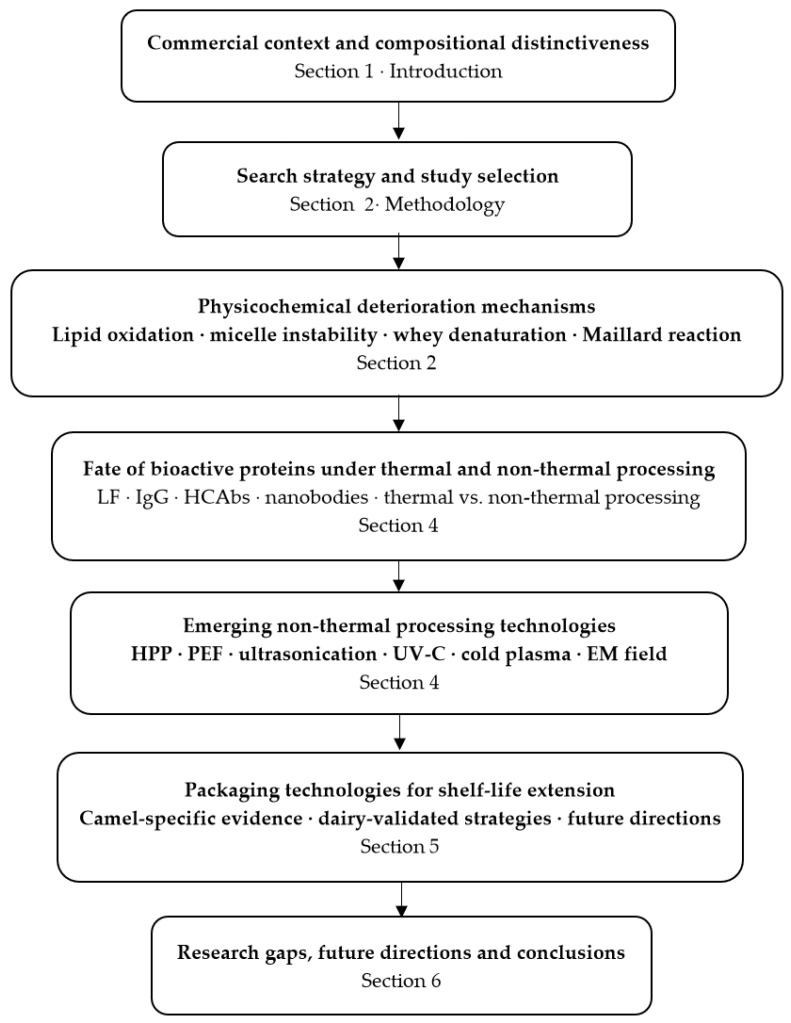
Review structure and conceptual flow.

CM, produced by *Camelus dromedarius* and *Camelus bactrianus*, has undergone an unprecedented change in the last 20 years, growing to be not only a commodity consumed regionally but also a globally recognized functional dairy product with a projected market value of USD 18.3 billion in 2027 [1,2]. Commercially, heat-treated CM products, including UHT-processed liquid milk and spray-dried powder, are now distributed internationally under brands such as Camelicious (United Arab Emirates), Desert Farms (United States), and QCamel (Australia), reflecting the sustained growth of global consumer demand for this commodity [3]. Nonetheless, despite this trade impetus, the scientific infrastructure supporting its shelf life management remains underdeveloped. The camel dairy sector still applies processing guidelines derived from BM models that are not directly applicable to the fundamentally different compositional profile of CM. At high temperatures, CM exhibits poor heat stability and cannot be sterilized at its natural pH due to the denaturation and sedimentation of proteins [4]. This thermal sensitivity is largely driven by the complete lack of β-LG, low κ-CN content, a unique CN micelle structure, and a whey protein (WP) composition that is fundamentally different from BM [5,6]. The nutritional uniqueness of CM—including its reduced allergenicity attributable to the complete absence of β-LG (the principal bovine milk allergen) and a casein fraction with substantially lower αs1-CN content, its high vitamin C level (4.80–5.95 mg per 100 g compared with approximately 2.0 mg per 100 g in BM), and higher levels of antimicrobial proteins—is what specifically contributes to its increasing commercial popularity [7,8]. But those are the very qualities that make it commercially valuable, which also present certain vulnerabilities when it comes to processing and storage. Balancing microbial safety with bioactive integrity, in the absence of validated processing protocols developed specifically for CM, is the central challenge of camel dairy shelf life science and the organizing problem this review addresses. That need is further heightened by the emerging uses of CM as a special nutritional product, such as sports nutrition, where the unique protein and electrolyte profile of CM has potential benefits for recovery and hydration, and as a clinical nutrition product, such as for immunocompromised populations, which require processing systems that maintain bioactivity but ensure safety [8]. Growing clinical interest also extends to neurodevelopmental conditions: randomized trials and meta-analyses of controlled studies have demonstrated significant reductions in childhood autism rating scores following CM consumption, an effect attributed to its casomorphin-free protein profile, antioxidant-rich composition, and immunomodulatory bioactive fraction, though larger-scale and longer-duration clinical trials are still required to establish definitive therapeutic recommendations [9,10]. The scientific infrastructure underpinning shelf life management must grow alongside this commercial trajectory, a development that, as it stands, is not yet adequately covered by the current evidence base. Recent publications have substantially advanced knowledge in this area: Hamed et al. (2024) examined the antimicrobial components of CM and fermented CM products, including shelf life considerations [11], while Rather et al. (2025) provided a comprehensive account of CM’s multitherapeutic potential [10]. Both contributions, however, did not systematically analyze the underlying physicochemical deterioration mechanisms associated with the specific compositional profile of CM, the fate of the most commercially valuable bioactive compounds during processing, and the potential of new non-thermal and packaging technologies tested in the CM matrix. The current review fills these related gaps in four thematic pillars, namely the physicochemical deterioration mechanisms, bioactive preservation under processing stress, emerging non-thermal technologies, and packaging innovations. The review framework is depicted in Figure 1. Throughout, BM serves as the reference species against which CM-specific processing behaviors and deterioration mechanisms are systematically compared, providing the species comparative framework that underpins the review’s analytical conclusions.

## 2. Materials and Methods

This narrative review was conducted in accordance with the Scale for the Assessment of Narrative Review Articles (SANRA) [12]. A structured literature search was performed across five electronic databases—PubMed/MEDLINE (*n* = 186), Scopus (*n* = 312), Web of Science (*n* = 278), ScienceDirect (*n* = 234), and Google Scholar (*n* = 468)—yielding 1478 records in total prior to deduplication, using Boolean combinations of the following terms: camel milk, shelf life, physicochemical deterioration, lipid oxidation, CN micelle, whey protein denaturation, Maillard reaction, LF, immunoglobulins, heavy chain-only antibodies (HCAbs), nanobodies, non-thermal processing, high-pressure processing, pulsed electric field, cold plasma, ultrasonication, UV-C irradiation, EM, active packaging, and shelf life modeling. No temporal restriction was applied to foundational mechanistic literature; publications from 2015 to 2026 were prioritized for emerging non-thermal technologies and packaging innovations. Eligible sources included primary research articles, systematic reviews, and peer-reviewed narrative reviews published in English. Reference lists of key included articles were hand-searched to identify additional relevant sources; gray literature and preprint repositories were not searched. Studies were excluded if they: (i) addressed only non-camelid dairy species without direct comparison with CM; or (ii) constituted conference abstracts or non-peer-reviewed reports. All quantitative claims were traced to their originating primary source; where direct primary validation was not possible, claims are explicitly cited as derived from secondary sources. Throughout the review, BM is used as the principal comparator species against which CM-specific composition, processing responses, and deterioration mechanisms are systematically evaluated, providing the species comparative framework that underpins the analytical conclusions of this review.

## 3. Physicochemical Deterioration Processes That Control CM Shelf Life

### 3.1. Compositional Profile of CM and Its Relevance to Shelf Life Vulnerability

The distinctive compositional profile of CM governs the characteristic pattern of its degradation during storage and processing. Recent meta-analyses have produced average gross compositional values for *Camelus dromedarius* and *Camelus bactrianus* CM of 3.17% protein, 3.47% fat, 4.28% lactose, 11.31% total solids, and 0.78% ash, alongside pooled micronutrient estimates of 112.93 mg/100 g calcium, 116.13 mg/100 g potassium, 53.10 mg/100 g sodium, 9.65 mg/100 g magnesium, 1.68 mg/100 g zinc, and 0.45 mg/100 g iron [13]. Compared with BM, CM has a similar gross protein content (3.17% vs. approximately 3.2–3.4%), lower total solids (11.31% vs. approximately 12.0–12.9%), lower fat (3.47% vs. approximately 3.5–4.0%), lower lactose (4.28% vs. approximately 4.6–4.8%), and lower cholesterol, alongside substantially higher iron (0.45 vs. approximately 0.04–0.05 mg/100 g), sodium (53.10 vs. approximately 44–50 mg/100 g), and vitamin C—pooled estimate of 5.38 mg/100 g (4.80–5.95 mg/100 g) versus approximately 2.0 mg/100 g in BM—and a pH typically ranging from 6.5 to 6.75 [13]. The relative resistance of CM to microbial growth when compared to BM can be explained by the composite antimicrobial protein system of the former, including LF (0.18–2.48 mg/mL), lactoperoxidase, and LZ [8,14].

The most significant compositional difference from BM is the protein system. CM whey is completely free of β-LG and contains α-lactalbumin (α-LA), camel serum albumin (CSA), PGRP, LF, LZ, and Igs [15]. In BM, thermal unfolding of β-LG above 65 °C drives sulphydryl–disulphide exchange reactions with κ-CN at the micelle surface, generating a heat-induced protein coat that substantially enhances colloidal stability under thermal and pH stress; the complete absence of β-LG in CM eliminates this stabilizing mechanism entirely, rendering CM inherently more susceptible to protein aggregation and colloidal destabilization under equivalent processing conditions [4,14,16]. A high β-CN to αs1-CN ratio, with significantly low κ-CN levels of approximately 3.3–3.5% of the total CN compared with about 12–13% in BM, determines the CN fraction, which is associated with poor coagulation behavior and a long rennet clotting time. The CN micelles of CM tend to be larger and more widely distributed in size than those of bovines, although absolute values differ across studies depending on analytical technique [17]. The fat globules of CM are smaller than bovine fat globules, measuring between 1.1 and 2.1 µm, as opposed to 1.6–4.9 µm in BM, and have a relatively thicker fat globule membrane [18]. The most diagnostically important quantitative differences between CM and BM that bear directly on shelf life behavior are summarized in Table 1. CM contains substantially higher concentrations of IgG, ranging from approximately 4.75 mg/mL in early lactation (day seven postpartum) to 0.7–2.2 mg/mL in mature milk [19], compared with 0.62–0.67 mg/mL in BM [20] as well as LF, ranging from 0.18 to 2.48 mg/mL compared with 0.08–0.5 mg/mL in BM [19], and LZ, present at 228–500 µg/100 mL versus approximately 11 µg/100 mL in BM [21,22]. These high levels have a combined effect in giving CM its inherently enhanced intrinsic antimicrobial defense. Remarkably, PGRPs are found in CM in a concentration of about 107 mg/L [23], and are completely absent in BM, offering another facet of intrinsic immune defense against Gram-positive microbes that is both processing sensitive and whose behavior under thermal and non-thermal conditions has received virtually no specific study despite its distinctive taxonomic distribution [8,23]. A very high concentration of vitamin C of 4.80–5.95 mg/100 g is significantly higher than the 2.0 mg/100 g in BM [24], which adds to an antioxidant effect that is quantifiably lost in thermal processing [1,7]. The level of iron is significantly higher at 0.29–0.53 mg/100 g as compared to 0.045 mg/100 g in BM [8], indicating a wider range of micronutrient density in CM. Most importantly, β-LG, which is a significant allergen in BM (4.4 g/L) and a key player in thermal processing reactions, is eliminated in CM [7], eliminating a key mediator of thermal processing responses. These quantitative dissimilarities are not just nutritional dissimilarities; they form the mechanistic foundation of the processing sensitivity and shelf life behavior defined during this review.

### 3.2. Lipid Oxidation

CM fat is a source of essential fatty acids, fat-soluble vitamins, and energy. The content of unsaturated fatty acids in it is relatively higher than in BM, a characteristic feature which provides nutritional benefit but, at the same time, exposes the lipid fraction to more oxidative degradation during storage and processing [25]. Short-chain fatty acids (carbons 4–12; butyric through lauric acid) are present in CM at six to eight times lower concentrations than in bovine, ovine, caprine, and buffalo milk, while the medium- and long-chain saturated fatty acids C14:0, C16:0, and C18:0, and the monounsaturated C16:1, predominate; the essential polyunsaturated C18:2n-6 (linoleic acid) is also present at comparatively elevated concentrations relative to BM [18,25]. This increased content of PUFA implies that CM fat globules are especially susceptible to peroxidation [18,25]. Lipid oxidation starts with the formation of hydroperoxides at the bis-allylic sites of linoleic (C18:2) and linolenic (C18:3) acids, followed by a cascade of reactions leading to aldehydes, ketones, and volatile compounds that cause rancid off-flavors; malondialdehyde (MDA) is the major measurable secondary oxidation biomarker used to assess lipid deterioration in processed milk systems [25].

Assays of 2,2-diphenyl-1-picrylhydrazyl (DPPH) radical scavenging activity by Mazumder et al. (2026) yielded antioxidant capacities of 62.4% ± 2.1% in raw CM, 60.8% ± 1.8% under EM treatment, 54.2% ± 2.5% following conventional thermal pasteurization, and 49.7% ± 2.2% after microwave treatment [26]. These authors noted that MDA measurements done using thiobarbituric acid-reactive substances (TBARS) were generally similar in thermally treated versus raw milk, which is an indication of retained bulk lipid stability; even the measurable decrease in antioxidant activity has a direct mechanistic implication for oxidative susceptibility during prolonged subsequent storage, as CM has compositional amplification of the PUFA oxidation substrate pool. This attenuation of antioxidant activity induced by processing is enhanced by thermal destruction of vitamin C: [26] recorded losses of about 23% during thermal pasteurization and about 25% following microwave treatment, which gradually removes natural oxidative activity at a time when the PUFA-rich fat fraction is most vulnerable to peroxidative injury. The interplay between depletion of the antioxidants and high levels of oxidative substrates is a synergistic risk of deterioration that is compositionally enhanced in CM compared to BM. The relatively low size of CM fat globules, although advantageous to digestibility, results in a higher overall interfacial surface area per unit volume of fat than in BM, a structural characteristic that promotes interface-mediated peroxidation [2,25,27]. Optimally performed thermosonication at 81 W power, for 5.9 min, at 41 °C resulted in a minimum size of fat globules in the dispersion and a maximum emulsion stability of 22.88% [25]. However, this smaller dispersion can also contribute to lipid oxidation when the antioxidant cover is diminished by thermal processing, creating a synergistic deterioration risk [25]. This oxidative vulnerability has direct implications for the choice of packaging: oxygen barrier packaging and headspace control are not optional but mechanistically required protective strategies for CM.

### 3.3. CN Micelle Destabilization and Colloidal Instability

The CN micelle organization of CM governs its colloidal stability in response to processing in markedly different ways from BM across all major processing intensities [7]. Camel CN micelles in the raw skimmed milk were 171.23 ± 4.18 nm in diameter, broader in distribution and larger than BM, and characterized by a markedly low κ-CN content of approximately 3.3–3.5% of total CN [7,17]. CM CN micelles were significantly reduced by 16.4% to 143.18 ± 2.34 nm following high-temperature short-time (HTST) pasteurization (72 °C, 15 s), whereas BM CN micelle size remained unchanged at 140.05 ± 2.29 nm [7]. CM CN micelles contracted further to 137.77 ± 1.52 nm—a 19.5% overall reduction relative to raw milk—under ultra-high temperature (UHT) treatment (140 °C, 5 s), while BM CN micelles counter-intuitively grew by 14% to 163.60 ± 3.70 nm under the same conditions [7]. This UHT difference can be explained by the fact that β-LG/α-LA complexes are formed under the influence of sulphydryl–disulphide exchange reactions at temperatures higher than 80 °C in BM; the complexes bind to the κ-CN-enriched micelle surface and lead to the observed swelling, which in CM is not possible due to the complete absence of β-LG [7]. It is proposed that this CM-specific size reduction pattern under HTST and UHT—in contrast to the size increase observed in BM—is attributable to dissociation of κ-CN from the micelle surface or to heat-induced precipitation of colloidal calcium phosphate from within the micelle matrix, both of which reduce rather than increase micelle size [7,28].

At HPP (200–800 MPa, 30 min, 20 °C), CM CN micelles again behaved differently: 200 MPa treatment resulted in a 21% decrease in CM CN micelles (135.22 ± 2.68 nm), whereas the same pressure caused only a 6% size decrease in BM CN micelles (134.90 ± 1.52 nm) [7]. CM CN micelles stabilized at 400–800 MPa showed only about 25% reduction from raw milk values (127–129 nm range), whereas BM CN micelles underwent much more drastic destabilization, with a 50% reduction to approximately the 71–73 nm range, driven by high-pressure disruption of hydrophobic and electrostatic interactions within the micelle interior [7]. The higher-pressure resistance of camel CN micelles is hypothesized to indicate their higher mineral content and the larger role of magnesium, inorganic phosphorus, and citrate in the micellar structure, forming more saline bridges that stabilize the colloidal structure against disintegration under pressure [7]. These mechanistically divergent colloidal behaviors between HTST, UHT, and HPP validate that CM CN behavior can no longer be forecasted using BM results at any processing intensity, and that there is a clear scientific need for camel-specific experimental models to guide processing protocol development. Heat-induced denaturation of WPs at higher temperatures (above 75 °C) in BM facilitates the association of denatured WP aggregates with the surfaces of CN micelles by the formation of WP κ-CN complexes, which block the reactive sites necessary to cause coagulation of the milk by rennet, resulting in a weaker coagulum [22,29].

The lack of β-LG in CM removes this particular pathway, but does not give the milk coagulation stability; instead, the colloidal susceptibility of CM to processing is due to the combination of very low κ-CN concentration, a coarse and more polydispersible CN micelle structure, changes in mineral balance during thermal stress, and a WP fraction that is highly sensitive to heat, particularly α-LA and PGRP, rather than to the β-LG/κ-CN disulphide exchange mechanism operative in BM [7,15]. The rennet coagulation time of raw CM was much lower than that of raw BM (16.16 ± 1.86 min versus 31.18 ± 1.07 min), indicating different chymosin cleavage kinetics of camel κ-CN, although the rennet coagulation properties of CM were substantially more sensitive to processing-induced disruption than those of BM [7].

This impairment of coagulation disproportionately after HTST is due to the aggregation of denatured WPs that block rennet-reactive sites [22,29]. HPP of 200 MPa increased the rennet coagulation time of CM to 25.20 ± 1.41 min, and HPP at 400 MPa increased it further to 33.84 ± 0.41 min; HPP of 600 and 800 MPa prevented CM coagulation. BM, in turn, did not lose coagulation activity at 600 MPa (rennet coagulation time 30.92 ± 1.86 min) and 800 MPa (32.09 ± 2.15 min), which highlights the fundamentally different pressure sensitivity of CM CN micelles compared with BM CN micelles [7]. In refrigerated storage, the production of lactate by microorganisms gradually acidifies the milk. CM stored at 4 °C loses protein, amino acid, fat, and fatty acid levels gradually over seven days, and also shows a significant increase in psychrobacter and a significant decrease in kocuria, which lead to a decrease in pH degradation of κ-CN, CN aggregation, and phase separation [27].

### 3.4. Whey Protein Denaturation Under Thermal Processing

Thermal processing of CM WPs is significantly different from that of BM, and the published literature exhibits real inconsistency because of biological and methodological variability, rather than experimental errors. Differential scanning calorimetry revealed that camel α-La has denaturation temperatures of 73.8 °C in rennet whey and 60.5 °C in acid whey and confirmed that camel α-La was the most heat-sensitive major WP in this species [30]. At 80 °C for 60 min, CSA concentration decreased by 42%, PGRP by 68%, and 100% of α-La disappeared from CM [31]. UHT treatment (140 °C, 5 s) produced the most pronounced WP denaturation in CM: α-LA underwent 65.55 ± 0.29% denaturation, followed by serum albumin at 12.58 ± 0.88% and LF at 3.65 ± 0.54% [7]. HTST pasteurization (72 °C, 15 s) yielded substantially lower denaturation: α-LA at 27.13 ± 3.23%, serum albumin at 2.97 ± 0.65%, and LF at 1.13 ± 0.51% [7]. HPP at 200 MPa induced lower denaturation than UHT across all fractions; increasing pressure from 400 to 800 MPa resulted in a significant progressive increase in α-LA denaturation up to 32.50 ± 2.05%, while serum albumin and LF remained notably pressure-resistant at 3.94 ± 0.07% and 2.93 ± 0.38% denaturation, respectively, considerably lower than those recorded under UHT conditions [7]. Critically, β-LG was absent from all CM samples but was present at 5.59 ± 0.26 mg/mL in raw BM, confirming that its absence is complete and structurally consequential [7].

Elagamy (2000) reported that camel WPs were more heat-resistant than their bovine counterparts across the 65–100 °C range [32], and work on dried camel whey has also hinted at a relatively higher heat sensitivity compared to liquid systems [33], highlighting that, in addition to thermal intensity, matrix state is a significant variable. The open molecular structure of camel α-La at low pH increases aggregation, which makes acid whey less thermally stable than sweet whey in CM. The lactation stage causes a significant change in the content of IgG from approximately 12.6 mg/mL in early milk to approximately 0.5 mg/mL three months postpartum [34,35], which alters the overall thermal behavior of the whey fraction. The breed of the camel, feeding schedule, geographic provenance, pH, calcium level, heat–time interaction, and the analytical endpoint all contribute to variability in reported results, and future research needs to report these variables systematically to allow cross-laboratory comparability.

### 3.5. Maillard Reaction and Non-Enzymatic Browning

The Maillard reaction is one of the most important mechanisms of quality degradation in CM, having the most harmful influence when it is subjected to UHT processing and long-term powder storage. The reaction unfolds in three mechanistically distinct stages: initiation by condensation of the lactose carbonyl group with free amino groups of lysine residues in milk proteins, yielding unstable Schiff bases and Amadori rearrangement products [36]; followed by an intermediate phase characterized by the formation of furfural derivatives, reductones, and reactive dicarbonyl compounds; and culminating in the advanced-stage generation of melanoidins directly responsible for brown discoloration, off-flavor development, and irreversible reduction in lysine bioavailability [36]. Furosine is a well-established marker of early-stage protein glycation, and hydroxymethylfurfural (HMF) is an indicator of advanced Maillard progression, both of which are recognized quality indices in heat-treated dairy products. Their specific thresholds and kinetic parameters have not, however, been validated for CM matrices, representing a meaningful gap in matrix-specific deterioration science that underpins evidence-based quality assurance for this commodity [37].

UHT treatment of CM generates advanced glycation end-products (AGEs) at concentrations substantially higher than HTST pasteurization, reducing the nutritional availability of essential amino acids, particularly lysine. At high relative humidity, spray-dried CM powder exhibits faster non-enzymatic browning proportional to water activity-dependent Maillard kinetics that is maximized at intermediate a*w* values of approximately 0.4–0.7 [38]. AGE accumulation has consequences beyond color change: it has been associated with decreased protein solubility, loss of powder reconstitution ability, and irreversible loss of functional protein activity, each of which directly diminishes the commercial value of CM [39]. An important practical implication is that the relatively low overall solid content of CM of about 11% (compared to 11.9% in BM [14]) and the unique mineral composition of CM both can affect the glass transition temperature of spray-dried powder and the rate of Maillard evolution in storage dynamics that bovine-derived powder models are unlikely to predict reliably [40].

The entire interplay of compositional uniqueness of CM, dynamics of glass transition, and kinetics of storage-induced Maillard reactions, however, is not fully characterized [4,40]. The β-CN predominance, making up about 65% of total CM CN versus about 39% in BM, together with its higher proline content and reduced capacity to form ordered secondary structure, may reduce the extent to which intermolecular crosslinking can occur between thermally exposed lysine residues and lactose, thereby partially suppressing the Amadori cascade relative to αs1-CN-dominated BM systems, though this mechanistic hypothesis remains untested in camel-specific matrices [4]. The ionic microenvironment might be partially affected by the characteristically increased sodium and potassium concentrations of CM [13], and this effect on Schiff base stability and reaction kinetics is unlikely to follow bovine-derived Maillard models, although this mechanistic hypothesis has not been experimentally tested in CM systems.

Moreover, the different whey–CN ratio changes the amount of thermally denatured WPs available to provide free amino groups to browning substrates during UHT treatment and spray drying; WPs tend to be more reactive Maillard substrates than intact CN micelles due to their more readily accessible lysine residues to thermal unfolding [7]. The formation of non-enzymatic browning and the development of Maillard reaction products have also been observed in powdered CM that has undergone the spray-drying process in accelerated storage, and their intensity improves with a higher inlet drying temperature [41], which confirms the practical importance of these reactions in determining the commercial shelf life of CM powder products. Dedicated investigation using validated AGE and furosine quantification methods is required, rather than extrapolation of bovine powder models.

### 3.6. Camel-Specific Modifications to Shared Dairy Deterioration Mechanisms

Dairy species-based mechanisms such as microbial acidification, lipid oxidation, non-enzymatic browning, and heat-induced protein denaturation operate in CM but fail to fully account for CM-specific processing challenges [1,11]. What distinguishes CM is a set of compositionally driven modifications to those shared pathways: the complete absence of β-LG and the deficiency of κ-CN remove principal molecular contributors to thermal colloidal stability, making CM intrinsically more susceptible to protein aggregation and sedimentation under standard pasteurization and sterilization conditions than BM [4,7]; the markedly low κ-CN content and larger, more polydisperse CN micelle organization create a protein matrix that responds to pressure, acid, and heat in ways that bovine dairy models do not reliably predict [7,17]. The smaller fat globules present a greater interfacial surface area per unit volume of fat, amplifying PUFA peroxidation risk [25]; and the composite antimicrobial protein system comprising LF, LZ, LP, Igs, and PGRPs, while providing genuine shelf life advantages under ambient conditions, is itself thermally sensitive, meaning that treatments required for microbial safety simultaneously degrade the very compounds responsible for CM’s natural protective capacity, creating a processing paradox with no direct bovine equivalent [8,10,32]. A structured synthesis of these camel-specific deterioration pathway modifications, their compositional drivers, and their practical shelf life implications is presented in Table 2.

## 4. Fate of Bioactive Proteins Under Thermal and Non-Thermal Processing

### 4.1. Bioactive Protein Composition and Functional Significance

CM possesses a remarkably rich bioactive protein fraction that distinguishes it from all other commercially relevant dairy milks. This fraction encompasses LF, LZ, LP, Igs (IgA, IgG, and IgM), PGRPs, and, uniquely among dairy species, HCAbs, whose single antigen-binding domains (VHHs or nanobodies) represent a structurally distinctive class of immunoproteins [8,10]. Camel Igs include conventional IgG and the unique HCAbs that naturally lack light chains; the HCAbs of IgG2 and IgG3 contribute approximately 75% of all serum IgG in camelids, are about one-tenth the size of conventional antibodies (15 kDa for the VHH domain), and possess high solubility, stability, and enhanced cell-membrane penetration ability [8,43].

### 4.2. Thermal Processing Effects on CM Bioactive Proteins

The thermal sensitivity of CM’s bioactive proteins represents the central tension in shelf life management. Elagamy (2000) demonstrated that pasteurization at 65 °C for 30 min preserved LF and LZ in CM without a significant effect, while IgG retained only 68.7% of its activity at 75 °C for 30 min, compared with complete loss of activity in bovine and buffalo milk IgG under the same conditions [32]. At 85 °C for 30 min, LF was completely inactivated in camel, bovine, and buffalo milk, while LZ lost 56% of its activity in CM, 74% in BM, and 81.7% in buffalo milk, demonstrating a comparative heat-resistance advantage for CM antimicrobial factors that is nonetheless partial and treatment-dependent [8,32]. Heating CM to 100 °C for 30 min caused complete inactivation of all antimicrobial factors in the milk, i.e., IgG, LF, and LZ, and made the milk microbiologically safe but inactivated its therapeutic effects [8,11,32].

The commercial implications follow a clear progression across treatment intensities. Low-temperature long-time (LTLT) pasteurization at 63–65 °C for 30 min preserves LF and LZ without significant effect and extends refrigerated shelf life to 7–20 days at 4 °C, though IgG activity is reduced by 31.3% at 75 °C for 30 min [8,10,11,32]. HTST at 72 °C for 15 s, the current industrial standard, limits LF denaturation to 1.13 ± 0.51% and extends refrigerated shelf life to more than 10–15 days at 4 °C, with moderate IgG loss and partial reduction in heat-sensitive vitamins [7,8,11]. High-temperature holding at 85–90 °C for 15–30 min achieves strong microbial reduction and improved viscosity in fermented product applications, but at the cost of complete LF loss and 56% LZ activity loss at 85 °C for 30 min [8,11,32]. UHT at 138–145 °C for 1–10 s delivers six to nine months of ambient shelf life but causes complete LF and PGRP denaturation, 65.55 ± 0.29% α-La denaturation, destruction of the majority of IgG, and off-flavor development [7,8,11,31]. In-container sterilization at 115–120 °C for 10–30 min achieves complete destruction of all spore-forming bacteria at the cost of total loss of all antimicrobial factor activity [8,11,32]. Among non-thermal approaches evaluated specifically for Ig effects, gamma irradiation at 9 kGy reduced total CM IgG by 13% without a significant effect on other major WPs, offering a more moderate Ig impact than UHT whilst achieving effective bacterial contamination reduction, though regulatory restrictions and public acceptance challenges constrain its practical adoption in most markets [8,11,44].

The CM antimicrobial factors hierarchy of heat resistance adheres to a uniform pattern among various independent studies: LZ > LF > IgG [8,32]. Notably, CM proteins retain a greater proportion of biological activity than their bovine and buffalo counterparts at equivalent thermal exposure, even when partial structural denaturation is evident. This species-level advantage has been attributed to the distinctive amino acid composition and glycosylation pattern of camel LF, which confer greater conformational resistance relative to bovine LF, and to the inherently high thermostability of HCAb single-domain structures, which lack the CH1 domain vulnerability present in conventional immunoglobulins [32,43]. This comparative advantage diminishes progressively but does not disappear with increasing treatment intensity [8,10]. This species-specific resilience offers a mechanistic basis for optimizing thermal processing parameters to take advantage of the innate benefits of CM without violating its eventual thermal limits. The implication in practice is that CM can be subjected to a more aggressive thermal regime before losing the same proportions of bioactive functionality as BM, but this benefit cannot be construed as permission to apply the same processing parameters used with BM without camel-specific confirmation [4,7,8].

The most complete comparative dataset available for these new technologies is quantitative evidence on EM and microwave preservation by [26]. Sodium dodecyl sulfate polyacrylamide gel electrophoresis (SDS-PAGE) analysis revealed that EM-treated samples (850 mT, three minutes) exhibited well-defined protein bands with minimal conformational change, while both conventional thermal pasteurization (TCP) and microwave treatments showed visible band attenuation. The degree of hydrolysis (DH%) was lowest in EM-treated milk (6.98 ± 0.23%) compared with TCP (11.05 ± 0.25%) and microwave (12.02 ± 0.29%). Enzyme-linked immunosorbent assay (ELISA)-based quantification showed LZ retention of approximately 87% under EM treatment versus 77% (TCP) and 73% (microwave); LF retention of approximately 77% under EM versus approximately 75% under both thermal comparators; and vitamin C loss of approximately 15% under EM treatment versus approximately 23% (TCP) and 25% (microwave) [26].

### 4.3. Non-Thermal Processing Effects on Bioactive Proteins: Evidence Summary and Research Gaps

The mechanistic profiles of each non-thermal technology—HPP, UV-C, ultrasonication, cold plasma, and EM treatment—are examined in Section 5; this section synthesizes their specific implications for CM bioactive protein retention.

Among all technologies evaluated, HPP at 200–400 MPa provides the most favorable bioactive retention profile in CM: LF denaturation at 200 MPa remains below 4%, and LF denaturation at 400–600 MPa (2.93 ± 0.38%) is substantially lower than the 3.65 ± 0.54% observed under UHT, confirming that LF is significantly better preserved under moderate HPP than under any thermal treatment [7]. The mechanistic basis for this resilience, in which the pressure-resistant CM CN micelle matrix may buffer co-localized WPs against pressure-induced unfolding, is detailed in Section 5.1.

The current evidence profile for the principal CM bioactive proteins across the full processing spectrum reveals critical gaps. LF shows no significant structural disruption under UV-C at 12.45 mJ/cm^2^ or under EM at 850 mT for three minutes, but its quantitative behavior during spray drying is not well documented [8,26]. LZ shows no significant loss under LTLT conditions and approximately 87% activity retention under EM conditions at 850 mT, but camel-specific HPP data for LZ are absent from the published literature [8,26]. For IgG, HPP-specific quantification in CM remains scarce, UV-C effects have not been evaluated, and spray drying substantially reduces IgG content depending on water activity and storage conditions [8]. PGRP remains the least characterized bioactive protein across all non-thermal conditions: quantitative data for HPP, UV-C, EM field, and spray drying effects in CM are entirely absent from the literature [8,31]. This evidence profile confirms that protein-specific, camel-validated investigation across the full processing spectrum is a priority research need.

### 4.4. The Nanobody Preservation Question

CM nanobodies (VHHs) are single-domain antigen-binding fragments derived from HCAbs, a class of Ig exclusive to camelid species. VHH domains represent the smallest naturally occurring antigen-binding fragments known to science, carrying a molecular mass of approximately 15 kDa and characterized by a long complementary determining region 3 (CDR3) loop capable of penetrating deeply within enzyme active sites, conferring a completeness of neutralization activity that conventional VH–VL paired antibodies rarely match [8,43]. The HCAbs from which VHHs are derived, principally the IgG2 and IgG3 subclasses, account for approximately 75% of total serum Ig in camelids, and their non-conventional single-chain architecture, which lacks both the light chain and the first heavy chain constant region (CH1), confers a degree of solubility and structural stability that conventional antibodies simply do not possess [8,45]. These biophysical properties have made camelid VHHs subjects of intense pharmaceutical and diagnostic interest, with well-documented applications spanning pathogen detection, targeted therapeutics, biosensor development, and the analytical chemistry of environmental contaminants [46,47].

The extent to which these stability benefits are meaningfully carried into maintained biological activity in the commercial CM processing environment, such as under heat gradients of HTST pasteurization, non-equilibrium pressure cycles of HPP, atomization and outlet temperature stress of spray drying, or generation of reactive species by ultrasonication and cold plasma, is altogether unexplained in the food science literature [8]. CM IgG retains only 68.7% of activity after pasteurization at 75 °C for 30 min, but bovine and buffalo milk IgG are fully inactivated under the same conditions—highlighting not only the excellent but imperfect thermal stability of camel immunoproteins but also the fact that processing has a measurable deleterious effect on bioactivity [8]. If nanobody-intact CM possesses different functional food properties, such as passive antimicrobial activity at mucosal surfaces and immunomodulatory activity, the lack of validated processing data will directly impede substantiated product claims and clinical application. The remarkable stability of VHH domains has been well defined in purified buffer systems [43,47]. But one should not think that this stability automatically applies to the whole CM. The native milk matrix consists of a much more complex physicochemical environment formed by continuous protein–protein interactions, mineral equilibria, fat globule membrane constituents, and progressive products of the Maillard reaction. It is worth mentioning that heat treatment of CM increases the levels of saccharide and dipeptides, which are precursors of the Maillard reaction, and consequently, changes the chemical environment of bioactive proteins [1]. The ultimate protection provided by these matrix-specific conditions that stabilize or destabilize VHH structural integrity under the conditions of processing stress is an open and significant question, one that deserves particular scrutiny before therapeutic assertions of processing-resilient VHHs can be made with assurance. In addition, nanobody aggregation propensity can be affected by the iron-binding status of co-present LF, and the ionic strength of the serum phase in a manner that cannot be predicted by pure system studies [8]. There is at least one commercially relevant gap in the science of CM processing that is currently known to lack any practical bioactivity measurement after processing, with most published research reporting gross protein denaturation instead of retained antigen-binding activity, and this gap is directly relevant to the functional food labeling, pasteurization of products, and the economic value of CM bioactivity [8]. To the extent that commercial processing conditions required to guarantee microbial safety simultaneously inactivate VHH binding activity, product labeling claims and pricing differentials based on HCAb content cannot be scientifically supported [32,43]. On the other hand, assuming that nanobodies can be proved to remain processing-stable at HTST conditions, e.g., because of their high thermal stability as reported, this would provide an extremely strong product differentiation case against bovine dairy versus CM. To answer this question, it is necessary: (i) to develop functional bioassays for measuring camel VHH activity in processed milk matrices; (ii) to systematically investigate the denaturation kinetics under HTST, UHT, HPP, UV-C, and spray drying conditions; and (iii) to correlate processing-induced structural changes with retained antigen-binding activity [43,45,48]. There is limited scientific evidence that VHH is maintained in commercial CM products, and until such studies are done, this information should be treated as scientifically unsubstantiated.

## 5. Emerging Non-Thermal Processing Technologies for CM

The choice of preservation technologies for CM cannot be made without reference to the mechanisms of deterioration described in Section 2. The small fat globule lipid fraction rich in PUFA in CM makes the case for low-thermal energy processing methods and prevents the formation of free radicals. This destabilization of camel CN micelles, which varies with pressure, is different from the destabilization of BM, and so HPP operating parameters based on BM might need to be recalibrated [7,18]. The thermolability of the bioactive protein fraction, especially α-La, PGRP, and LF, supports the view that non-thermal methods, where microbial safety targets can be attained without the temperature-dependent denaturation cascade, are favored. These mechanistic relationships should not be based on efficacy standards alone, but on technology choice [10,26,32].

### 5.1. High-Pressure Processing (HPP)

HPP is a process by which milk is processed at pressures of 200–800 MPa at 0–40 °C for a time ranging from several seconds to 20 min [49]. As established in Section 3.3, CM CN micelles exhibit only approximately 25% size reduction at 400–800 MPa compared with approximately 50% fragmentation in BM, confirming that bovine HPP models cannot be applied to CM [1,7]. HPP within the 400–600 MPa range and a one- to five-minute exposure window achieved pathogen reductions of 5 log_10_ CFU/mL *against L. monocytogenes, E. coli,* and *Salmonella* in CM studies [10], while effects on WP denaturation and color change were considerably less pronounced [7,8].

A particularly notable camel-specific finding from Aljasass et al. (2023), who subjected CM samples to HPP at 200–600 MPa and 40 °C for five minutes, is that treatment at 300 MPa reduced the total bacterial count to below 10^3^ CFU/mL, with this reduced load remaining stable for 15 days under refrigeration at 3 °C, whereas untreated samples reached spoilage levels within approximately one week [50]. Critically, HPP at this pressure range did not significantly affect the chemical composition, color, fat oxidation, pH, or organoleptic characteristics of CM [51]. However, a distinctive phenomenon exclusive to CM, not observed in bovine or goat milk under identical conditions, was identified: milk clotting occurred in samples treated at pressures at or above 350 MPa [50]. This clotting threshold appears linked to CM’s distinctive protein and mineral composition and represents a practically significant upper pressure constraint in HPP protocol design, as clotting reduces the bactericidal efficacy of pressure treatment [50]. Additionally, HPP reduced the proteolytic activity of CM, with treatments at 250 and 350 MPa significantly reducing protease content from 4.23 to 3.61 and 2.98 µM/mL, respectively, offering the additional benefit of delaying proteolysis-driven spoilage during refrigerated storage [50]. HPP thus represents the strongest near-term, mechanistically validated non-thermal option for CM, though the high capital cost remains the principal barrier for smaller-scale operations in camel producing regions [8].

### 5.2. Pulsed Electric Field Technology

Pulsed electric field (PEF) technology is the direct method of treating food material by applying short-duration and relatively low-energy input pulses of a repetitive electric field to food located between two electrodes in a treatment chamber, whereby the natural ionic conductivity of the food material enables the transfer of the pulse [1]. PEF treatments of 15–40 kV/cm have been shown to be effective in microbial inactivation in mixed dairy systems, preserving the integrity of vitamins and the stability of angiotensin-converting enzyme inhibitory peptides during storage, and having minimal sensory effects compared with conventional pasteurization [52]. PEF has a variable level of enzyme inactivation, which is determined by the source of the enzyme and the intensity of treatment [53]. Of particular interest to the lipolytic shelf life risk of CM, PEF has been found to reduce lipase activity by progressive increments in pulse number and the strength of the electric field [54]. When PEF intensity is increased, the size of the milk fat globules is decreased, and their total specific surface area is enhanced [55], an effect that could be either positive or negative based on the profile of concomitant oxidative exposure of the matrix. PEF treatment and milk protein functionality are not well defined due to the limited number of published studies on the PEF impacts on milk components [56]. None of the studies devoted to CM treatment with PEF have been reported in the primary literature; the existing treatment and compositional information have been obtained only from the bovine and mixed dairy systems [1]. Its appearance here is due to the proven applicability of the technology to the wider dairy processing and its likely applicability to CM, rather than its proven performance in camel-specific experimental systems. Even a single PEF study focused on camels alone is an important evidence gap that should be regarded as a high-priority research need, especially since the technology has a relatively low thermal load and can be used to preserve LF and IgG [1].

### 5.3. Ultrasonication and Thermosonication

Among non-HPP non-thermal technologies, ultrasonication has the most developed experimental evidence base from studies conducted specifically on CM [1]. Sonication at 30 kHz facilitates partial denaturation and aggregation of CN and WPs by acoustic cavitation, decreases the size of fat globule particles, enhances the negative charge on the surface of CN micelles, and achieves a 4.4-log_10_ reduction in *S. typhimurium* and the total inactivation of *E. coli* O157:H7 after 15 min of treatment.

Specific thermosonication of CM cream determined that thermosonication at 81 W, 5.9 min, and 41 °C resulted in the smallest fat globules and maximum emulsion stability of 22.88% at an energy density of 285 kJ/L [25]. There are two camel milk-specific constraints that need to be stated. To begin with, high-intensity sonication is reported to destabilize LF and LZ, which are the most commercially important bioactive compounds in CM [10]. The acoustic cavitation effect causes localized transient hotspots and shear forces, which may cause denaturation of the protein tertiary structure, and the danger is magnified by the cysteine-rich LF molecule, whose iron-binding and antimicrobial activities rely upon an intact tertiary conformation [10,57,58]. Second, ultrasonication produces free radicals by causing water dissociation and cavitation in the fat matrix of CM; in the PUFA-rich surface of the fat matrix, these radicals can induce and enhance lipid peroxidation, which can in turn reduce instead of extending oxidative shelf life unless the intensity or duration of treatment is monitored closely [10,57]. Both dangers can be alleviated by thermosonication under optimized conditions, whereby power, temperature regulation, and exposure duration are moderated, yet the parameter optimizations associated with CM product formats are still waiting to be performed on an industrial scale.

### 5.4. Ultraviolet Radiation

Microorganisms are inactivated by UV-C radiation (200–280 nm) by direct absorption of photons by nucleic acids, forming cyclobutane pyrimidine dimers and causing the formation of double-strand DNA breaks, which render the cell unable to replicate, a fundamentally non-thermal process that is inherently compatible with the preservation of heat-sensitive bioactive proteins [59]. In skimmed CM at 12.45 mJ/cm^2^, UV-C treatment achieved 3.9-log_10_ reductions in both *E. coli* O157:H7 and *S. typhimurium* without significantly affecting the protein electrophoresis pattern, confirming that protein denaturation is minimal at the doses effective against common pathogens [1,8]. The European Food Safety Authority has noted that UV treatment of milk can increase vitamin D3 concentration through photochemical conversion of 7-dehydrocholesterol, a potentially beneficial side effect given CM’s role as a functional food in populations with limited solar exposure [59]. However, UV-C treatment induced three new volatile compounds in CM: p-cresol, octanoic acid, and tetradecanal, whose sensory impact in whole milk formulations has not been systematically evaluated. p-Cresol in particular is associated with barnyard off-flavors in dairy products and may represent a consumer acceptability challenge even at sub-threshold concentrations over refrigerated shelf life [59]. More critically, the limited penetration depth of UV-C radiation in turbid, fat-containing matrices, where light scattered by fat globules and CN micelles attenuates effective fluence within millimeters of the liquid surface, remains the most significant technical barrier to industrial adoption [1,59]. The reported efficacy data are drawn exclusively from skimmed CM; validation in whole milk at field-representative microbial loads and product geometries is absent from the literature.

### 5.5. Cold Plasma Technology

Cold plasma, also known as non-equilibrium atmospheric plasma or non-thermal plasma, occurs when gas is exposed to enough electrical energy to ionize a portion of its constituent molecules to form a reactive mixture of electrons, photons, positive ions, excited neutral species, and reactive oxygen and nitrogen species (RONS) such as hydroxyl radicals (OH) and atomic oxygen (O) ozone (O3), nitric oxide (NO), and hydrogen peroxide (H_2_O_2_) [60]. Three main mechanisms are responsible for microbial inactivation: physical etching and peroxidation of the cell surface by reactive species, volatilization and desorption of cellular compounds due to UV photon bombardment, and oxidative damage to nucleic acids that causes irreversible genetic damage [1]. The three main types of cold plasma generators considered in food processing applications include dielectric barrier discharge (DBD), plasma jet, and gliding arc discharge. Cold plasma has also shown a wide spectrum of antimicrobial activity in bovine and ovine milk systems at temperature conditions close to ambient temperature, and it can be used intrinsically with bioactive proteins that are sensitive to heat [1]. The first systematic investigation of DBD cold plasma applied directly to CM was reported by Keewan et al. (2026), who employed a custom-built DBD reactor under optimized conditions (42 kV, 40 mm electrode gap, 15 min, M65 gas mixture: 65% O_2_ + 30% CO_2_ + 5% N_2_), achieving greater than 99.9% reduction in total bacterial count while maintaining a milk surface temperature below 29 °C, confirming genuine non-thermal operation [61]. Under these conditions, DBD-CP retained 92.6% of total protein, 91.3% of reducing sugars, 91.1% of triglycerides, 94.8% of vitamin D, and 81.4% of ascorbic acid relative to raw CM, retention profiles uniformly exceeding those of conventional pasteurization at 72 °C for 15 s [61]. DBD-CP maintained more than 85% of LF content, while thermal treatment caused approximately a 42% loss; IgG retention exceeded 90% following plasma treatment versus approximately a 33% loss after pasteurization; and insulin concentration remained above 90% of raw milk levels compared to a 31% reduction under heat [61]. Fourier transform infrared spectroscopy (FTIR) analysis also confirmed that no lipid structural degradation was observed, and typical C=O stretching and CH2 bending peaks were also not changed. TBARS analysis showed that lipid peroxidation was not detected following treatment of plasma [61]. Refrigeration increased shelf life fourfold, and treated samples had a shelf life of 16 days versus 4 days in untreated raw milk [61]. These results collectively establish DBD-CP as a technically viable non-thermal preservation platform for CM. However, the study was conducted at bench scale using milk from a single herd at a specific lactation stage, and camel-specific data on the effect of RONS on PUFA oxidative stability across extended storage, LF iron-binding activity under different plasma gas chemistries, and IgG immunoreactivity at the functional epitope level remain to be characterized before processing recommendations can be responsibly generalized across commercial production conditions [61].

### 5.6. Electromagnetic Field (EM) Processing

EM processing has emerged as a promising non-thermal approach for CM preservation, uniquely supported by direct camel-specific experimental evidence. Mazumder et al. (2026) evaluated EM treatment at 850 mT for three minutes, comparing it with microwave treatment (250 W, 30 s) and conventional thermal pasteurization [26]. Microbial inhibition increased progressively with field strength: 100 mT and 200 mT achieved reductions of only 0.05 and 1.03 log_10_, respectively; 400 mT produced a 1.30-log_10_ reduction; and 600 and 800 mT achieved 2.39 and 2.53 log_10_ reductions, respectively [26]. The optimal condition of 850 mT for three minutes reduced the initial load of 6.8 × 10^6^ CFU/mL to 1.5 × 10^4^ CFU/mL, a 2.66-log_10_ reduction (99.78% microbial reduction), within the microbiological limits reported in that study. Microbial load monitoring over 15 days of refrigerated storage confirmed extended shelf life, and sensory evaluation confirmed that EM-treated milk achieved the highest overall acceptability score (8.0 ± 0.65) compared with TCP (7.7 ± 0.73) and microwave-treated milk (7.1 ± 0.69), with significant differences in smell, consistency, and taste [26].

The probable mechanism involves electroporation of microbial cell membranes and oxidative stress mediated by field-driven free radical generation, both of which selectively target microbial cells rather than the larger protein and lipid structures of milk [26,62,63]. EM treatment consistently outperformed both microwave treatment and conventional thermal pasteurization in bioactive protein retention: LZ was preserved at approximately 87%, LF at approximately 77%, and IgG at 81% of baseline concentrations, compared with 65% IgG retention under thermal treatment [26]. The degree of protein hydrolysis, determined by the OPA assay, was lowest under EM treatment at 6.98 ± 0.23%, versus 11.05 ± 0.25% for thermal and 12.02 ± 0.29% for microwave conditions [26]. Vitamin C showed only approximately a 15% reduction under EM treatment compared with approximately 25% and 23% under microwave and thermal conditions, respectively, while vitamin D remained stable across all treatments [26]. TBARS analysis confirmed that EM treatment did not induce significant lipid peroxidation relative to raw CM [26]. Both EM and microwave treatments extended refrigerated shelf life to 15 days at 4 °C, compared with spoilage of untreated raw milk by day 6 [26].

Several important limitations must be acknowledged. The reported 2.66-log_10_ reduction, while meeting the microbiological thresholds cited by the authors, falls below the 5-log_10_ pathogen reduction target required in several regulatory jurisdictions for pasteurization equivalence claims [26]. The absence of multi-strain microbial validation, particularly against spore-forming pathogens and heat-resistant spoilage organisms, and the lack of industrial-scale evaluation significantly limit immediate translational applicability [26]. The parameter space of EM treatment, including field intensity, exposure duration, pulse frequency, and chamber geometry, has not yet been systematically optimized for CM across product formats or microbial challenge conditions [62], and independent replication by laboratories other than the reporting group is necessary before EM treatment can be considered validated at the level of HPP [26]. Most critically, the question of whether commercially applied pasteurization conditions inactivate VHH nanobody antigen-binding activity in CM remains entirely unanswered; until validated functional bioassays in processed CM matrices are established and systematically applied, product-labeling claims and pricing premiums based on HCAb content cannot be scientifically substantiated.

### 5.7. Comparative Performance of Non-Thermal Technologies in CM

A comparative evaluation of the principal non-thermal preservation technologies against consistent criteria is presented in Table 3. Of the technologies evaluated, HPP at 200–400 MPa currently represents the most commercially viable near-term option for CM: it is supported by the strongest camel-specific mechanistic evidence base, is formally approved for food processing in multiple regulatory jurisdictions, and is increasingly accessible to medium-scale operations through contract processing services provided by specialist HPP manufacturers such as Hiperbaric (Burgos, Spain) and Avure Technologies (Middletown, OH, USA) [10,49].

## 6. Packaging Technologies for CM Shelf Life Extension

The packaging strategies for CM continue to be mainly extrapolative as opposed to processing technologies, with little camel-specific validation [11,64]. It is not only an academic constraint, but also a real commercial risk, since the compositional differences between CM and bovine dairy products imply that the oxidative, microbial, and sensory stresses that this product experiences during distribution might not be similar to those faced by bovine dairy products, and thus package design decisions based on bovine experience cannot be projected onto CM [11,65].

### 6.1. Available Evidence on CM Packaging and Cold Chain Distribution Challenges

Specifically, Alshikh et al. (2021) examined how various packaging materials affect the shelf life of pasteurized CM and determined that the choice of packaging material has a strong impact on microbial growth dynamics, oxidative degradation, and sensory deterioration of pasteurized CM during refrigerated storage [64]. The study is the first, and in fact the only, camel-specific evidential anchor for packaging science in this area, and its conclusions must be interpreted within that framework: the terrain of CM packaging science is currently constructed on a very thin primary evidence base. Pasteurized CM has a shelf life of 10 to 15 days at 4 °C under refrigerated storage [10,42], and the shelf life of both raw and pasteurized CM at ambient and refrigeration temperatures is longer than that of BM, with acidity development proceeding more slowly owing to the composite antimicrobial protein system. This natural benefit alone, however, does not meet the long distribution chain and ambient shelf life demands of global market expansion, especially given cold chain reliability issues that typify the CM supply chains of remote arid and semi-arid producing areas [11,65]. Cold chain malfunctions are especially harmful since temperature abuse is a concomitant cause of microbial growth, lipid oxidation, and proteolysis, all of which occur more quickly in CM compared to BM due to its rich fat fraction of PUFA and reduced buffering capacity against pH-driven destabilization of CN micelles [27].

### 6.2. Dairy-Validated Packaging Strategies with Plausible Relevance to CM

Active packaging systems that include antimicrobial agents, oxygen scavengers, or moisture regulators are also approaches that have proven dairy performance histories and rational scientific bases for application to CM [66]. Of special mechanistic interest are oxygen-scavenging films: the larger proportion of PUFA in CM and the smaller size of fat globules are the source of an inherently greater susceptibility of CM to oxidative rancidity, and passive barrier packaging, the industry standard today, does not provide any active interventions against the hydroperoxide formation cascade once oxygen has entered the headspace [25]. Active oxygen scavenging is already commercially available to give long shelf life to bovine dairy products and can be applied to CM products to give measurably improved shelf life, but the quantitative advantage in terms of accumulation of MDA and sensory threshold increase has not been experimentally established in CM systems [64]. Natural agent antimicrobial packaging films (including nisin, chitosan, or plant-derived essential oils) may be considered as a complement to the endogenous antimicrobial protein system of CM as a supplementary, processing-independent barrier, especially in products in which UHT or HPP has partially destroyed the inherent antimicrobial fraction [11,66]. Additional essential oils such as spearmint and wild thyme have been directly added to CM, which positively affects shelf life and organoleptic properties [11,67]. Incorporation of such compounds into packaging matrices as sustained-release liners, as opposed to direct milk additives, provides a cleaner-label packaging science adaptation, which should be evaluated specifically for CM [64]. In particular, intelligent packaging systems such as time–temperature indicators, pH-sensitive color sensors, and freshness indicators sensing volatile signs of fat oxidation or microbial spoilage are applicable to CM due to the cold chain susceptibility of distant production supply chains, where temperature misuse can cause products to become hazardous long before the label date [64]. Although their performance in the dairy context has been reported, their individual calibration to CM spoilage indicators, such as MDA thresholds and volatile profiles of CM-specific spoilage microbes, has not been done yet [27]. Table 4 provides a systematic survey of packaging technologies, their mechanistic justification, evidence level, and research agenda.

### 6.3. Hypothetical and Early Concept Opportunities for CM

Films made of CM whey, which is devoid of β-LG but contains LF and LZ, would, in theory, create films with inherent antimicrobial activity, providing a model of the circular economy in the camel dairy business and a truly differentiated packaging product [68]. This antimicrobial effect would be based on the reported activity of LF and LZ against usual dairy spoilage and pathogenic microorganisms, although this activity had not been previously determined for this type of polymeric protein film. Another potential candidate is chitosan-based antimicrobial films that can be used in packaging dairy products [11,68]. They are research hypotheses, not solutions; no published primary research has proven the fabrication, characterization, or shelf life performance of camel whey-based packaging films, and these directions should be conveyed as research priorities to be experimentally validated, not as viable near-term alternatives to be adopted by the industry [11,68].

## 7. Research Gaps and Future Directions

The greatest gap is the lack of standardized kinetic shelf life models that have been constructed explicitly around CM products. Today, shelf life forecasts are either non-existent or based on BM data, a method that is scientifically invalid since the compositional variations between this review and past studies are fundamentally different [11]. The most pressing methodological priority is validated predictive models that would consider the special lipid oxidation kinetics, protein denaturation rates, and microbial growth parameters in specific storage conditions for CM [3]. The second gap is the systematic characterization of bioactive proteins, specifically the stability of HCAbs under commercial processing [3]. This gap will directly impact product quality assurance, functional food labeling, and the development of processing procedures that maximize the value retained in the final product. The nanobody preservation question represents perhaps the most commercially consequential unanswered question in CM food science. The third gap is the complete absence of life cycle assessment studies connecting preservation technology choices for CM to quantified sustainability outcomes [3]. As the camel dairy industry scales globally, the environmental credentials of different processing pathways require quantification to support evidence-based development decisions. A fourth priority gap with direct public health implications is the absence of validated camel-specific pasteurization verification markers. Alkaline phosphatase, the standard bovine indicator of pasteurization adequacy, remains active in CM following conventional HTST treatment, rendering it unsuitable for safety verification in camel dairy processing and creating a regulatory blind spot that urgently requires dedicated investigation [8]. Additional gaps warranting systematic investigation include the optimization of combined processing approaches (hurdle technology) calibrated specifically for CM; validation of active and intelligent packaging systems for CM’s unique chemistry; and dedicated PEF, cold plasma, and UV-C studies in whole CM.

## 8. Conclusions

This review has examined three interconnected dimensions of CM shelf life science that the existing literature has left substantially unaddressed: the physicochemical mechanisms of deterioration, the processing-induced fate of bioactive proteins, and the application of non-thermal and packaging technologies to CM preservation. The central thesis developed from this synthesis is that the compositional uniqueness of CM simultaneously confers inherent antimicrobial benefits and induces specific processing vulnerabilities when conventional BM protocols are applied. This presents the greatest technological challenges in camel dairy shelf life science. Among non-thermal technologies, HPP at moderate pressures (200–400 MPa) currently represents the strongest near-term candidate, offering the most mechanistically validated and camel-specific evidence base for microbial inactivation with acceptable protein and quality retention. EM processing at 850 mT for three minutes was reported in a primary CM study to achieve microbial control with retention of approximately 87% LZ activity, 77% LF, and the highest IgG retention among the treatments evaluated, alongside the highest sensory acceptability scores, genuinely promising findings that nonetheless derive from a single study and require independent replication, including multi-strain microbial validation and industrial-scale evaluation, before EM treatment can be considered validated at the level of HPP. PEF, UV-C, and ultrasonication each offer partial camel-specific support but require further camel-dedicated experimental investigation before confident positioning. Cold plasma has received its first systematic camel-specific investigation through DBD treatment [61], with promising results in bioactive retention and microbial reduction, though bench-scale limitations and the absence of independent replication mean that definitive conclusions cannot yet be drawn.

A complementary and processing-independent layer of protection is offered by functional packaging systems that actively control the oxygen, moisture, and microbial environment surrounding CM. The evidence base for CM packaging in particular is rather limited, as the study by Alshikh et al. (2021) remains the main focus, and the limited packaging evidence base must be clearly acknowledged in any claims made about CM packaging performance [64]. The increasing evidence base reviewed here is indeed encouraging, and the field direction is evident. Nevertheless, the discipline has reached the stage where no more descriptive work can be done, and significant advancement is to be made by controlled, camel-specific experimental validation of integrated processing, packaging, and storage systems—research should be conducted with the urgency dictated by the commercial path of the global camel dairy industry.

## Figures and Tables

**Table 1:** Key compositional parameters of dromedary CM and BM relevant to shelf life behavior.

Parameter	CM	BM	Shelf Life Relevance
**Total protein (%)**	3.17	3.2–3.4	Similar gross content; fraction composition fundamentally different (β-LG absent, lower αs1-CN in CM)
**Fat (%)**	3.47	3.5–4.0	Comparable; CM fat globules smaller (1.1–2.1 µm vs. 1.6–4.9 µm) with higher PUFA → amplified oxidative risk
**Lactose (%)**	4.28	4.6–4.8	Lower in CM; reduces Maillard substrate pool relative to BM
**Total solids (%)**	11.31	12.0–12.9	Lower in CM; affects glass transition temperature and Maillard kinetics in CM powder
**Vitamin C (mg/100 g)**	5.38 (4.80–5.95)	~2.0	~2.7-fold higher in CM; key antioxidant buffer substantially reduced by thermal processing
**Iron (mg/100 g)**	0.45	0.04–0.05	~9-fold higher in CM; pro-oxidant cofactor amplifying PUFA peroxidation risk
**pH**	6.5–6.75	6.5–6.80	Comparable range; CM more susceptible to pH-driven CN micelle destabilization due to low κ-CN
**β-LG**	Absent	~4.4 g/L	Absent in CM; eliminates the β-LG–κ-CN thermal stabilizing mechanism operative in BM
**κ-CN (% of total CN)**	3.3–3.5	~12–13	~4-fold lower in CM; impairs heat stability, rennet coagulation, and colloidal resistance
**LF (mg/mL)**	0.18–2.48	0.08–0.5	Substantially higher in CM; primary antimicrobial factor; heat-sensitive above 75 °C
**IgG (mg/mL)**	0.7–2.2 (mature)	0.62–0.67	Higher in CM; includes HCAbs absent in BM; thermally vulnerable above 75 °C
**LZ (µg/100 mL)**	228–500	~11	20–45-fold higher in CM; comparatively heat-resistant antimicrobial factor
**PGRP (mg/L)**	~107	Absent	Unique to CM; processing behavior under all treatments largely uncharacterized

CM values are pooled estimates from global meta-analysis [13] unless otherwise stated. BM comparator values are indicative reference ranges. IgG values reflect mature milk; early-lactation CM values reach approximately 4.75 mg/mL [19]. Sources: [7,8,13,14,17,19,20,21,23].

**Table 2:** Camel-induced adaptations in common dairy degradation mechanisms: compositional and shelf life effects.

Deterioration Pathway	Shared Dairy Mechanism	Camel-Specific Modification	Primary Compositional Driver	Shelf Life Implication
**Lipid oxidation**	PUFA hydroperoxide formation; secondary oxidation to aldehydes, ketones, and volatiles	Accelerated interface-driven peroxidation due to elevated surface-area-to-volume ratio of fat globules	Smaller fat globules (1.1–2.1 µm vs. 1.6–4.9 µm in BM); higher PUFA content	Greater risk under standard storage; oxygen barrier packaging is mechanistically necessary, not optional
**CN micelle destabilization**	Heat- and acid-induced aggregation and sedimentation	Micelles decrease in size under HTST and UHT (opposite to bovine behavior); HPP causes progressive size reduction without disintegration; coagulation inhibited above 600 MPa HPP	Absence of β-LG–κ-CN interaction; low κ-CN content (~3.3–3.5% vs. ~12–13% in bovine); larger, more polydisperse micelle organization	Bovine pasteurization models inapplicable; elevated aggregation and sedimentation risk under conventional thermal conditions
**Whey protein denaturation**	Heat-induced unfolding and irreversible aggregation	α-La most heat-labile (Td 60.5–73.8 °C depending on whey type); complete α-La loss at 80 °C/60 min; no β-LG thermal buffer present	Complete absence of β-LG; distinct whey fraction composition	Greater denaturation of heat-labile components under bovine-calibrated HTST conditions; bioactive fraction more thermally vulnerable than bovine equivalents
**Maillard browning**	Lactose–lysine condensation to Amadori products and melanoidins	Higher proline content in β-CN may partially attenuate Amadori cascade; ionic microenvironment effects on Schiff base kinetics incompletely characterized; whey-to-CN ratio shifts browning substrate pool	High β-CN-to-αs1-CN ratio; elevated Na^+^/K^+^ concentrations; distinct whey-to-CN ratio; low total solid content (~11% vs. ~12% in bovine)	Bovine-derived browning models not validated for CM powder; AGE accumulation and lysine loss may differ substantially from predicted rates
**Microbial acidification**	Microbial lactate production → pH decline → κ-CN depletion → CN aggregation	Natural antimicrobial system (LF 0.18–2.48 mg/mL; LZ 228–500 µg/100 mL; IgG 0.7–2.2 mg/mL in mature milk; LP; PGRPs) provides intrinsic resistance; acidity development slower than in BM	Composite antimicrobial protein system acting in concert; substantially higher LF and LZ than BM	Extended natural shelf life under ambient conditions; however, antimicrobial system is heat-sensitive and partially inactivated by pasteurization, creating a processing paradox

Mechanisms marked in the Maillard browning row (proline-mediated Amadori attenuation; ionic microenvironment effects on Schiff base kinetics) represent mechanistic hypotheses that have not been experimentally validated in CM matrices. Bovine comparator values for fat globule size (1.6–4.9 µm), κ-CN content (~12–13%), and total solids (~12%) are derived from Hailu et al. (2016) and Seifu (2023). Source: [1,7,8,11,14,17,25,30,31,42].

**Table 3:** Microbial efficacy, bioactive retention and strength of evidence of *CM* non-thermal technologies compared.

Technology	Microbial Efficacy	Bioactive Retention	Physicochemical Stability	Sensory Impact	Strength of Camel-Specific Evidence	Scalability	Key Limitations
**HPP (200–400 MPa)**	High; 5-log_10_ pathogen reduction in camel-milk studies	Moderate to high; LF denaturation 2.93% at HPP; α-La 32.5% at 400–600 MPa; considerably less than UHT	CN micelle size reduction; coagulation inhibition above 600 MPa	Minimal under moderate pressures	Strongest current camel-specific evidence base	Moderate; high capital cost limits small-scale adoption	High equipment cost; inhibits coagulation above 600 MPa
**Pulsed Electric Field**	Moderate in bovine dairy; no camel-specific data	Good retention in bovine systems; camel data absent	Fat globule size reduction at high intensity	Minimal in bovine systems	Absent; entirely extrapolated from bovine dairy	Moderate; complex equipment	No camel-specific evidence; cannot be recommended without dedicated studies
**Ultrasonication**	Moderate; 4.4-log_10_ *S. typhimurium* and complete *E. coli* O157:H7 inactivation at 900 W/20 kHz/15 min	Limited at high intensity; LF and LZ destabilization in CM reported	Reduces fat globule size; improves emulsion stability	Minimal under controlled conditions	Moderate; multiple camel-specific data points available	Good; relatively accessible	Bioactive destabilization; PUFA lipid oxidation risk
**UV-C Radiation**	Moderate; 3.9-log_10_ reductions at 12.45 mJ/cm^2^ in skimmed CM	Protein structure largely preserved; 3 new volatiles (p-cresol, octanoic acid, tetradecanal)	Minimal effect on protein electrophoresis	Potential off flavors; needs validation in whole milk	Limited to skimmed CM only	Constrained by penetration depth in whole milk	Below 5-log_10_ regulatory target; penetration limits whole milk
**Cold Plasma (DBD)**	>99.9% reduction in total bacterial count at 42 kV, 40 mm electrode gap, 15 min; milk surface temperature maintained below 29 °C, confirming genuine non-thermal operation	>85% LF retention vs. ~42% loss under pasteurization; IgG retention >90% vs. ~33% loss after pasteurization; insulin >90% of raw milk levels; 81.4% ascorbic acid and 94.8% vitamin D retained	No lipid structural degradation confirmed by FTIR; no measurable lipid peroxidation by TBARS analysis	Not systematically evaluated in the primary study	Limited to one bench-scale camel-specific study using a single herd at a specific lactation stage; requires independent replication	Bench scale only; industrial-scale scalability not yet established	Single study; single herd; RONS effects on PUFA over extended storage uncharacterized; LF iron-binding activity under different gas chemistries not evaluated; IgG immunoreactivity at epitope level undetermined
**EM (850 mT)**	2.66-log_10_ reduction; 99.78% microbial reduction in the available primary study	Approximately 87% LZ, 77% LF retained; DH% 6.98 ± 0.23% in the available study	Minimal protein denaturation confirmed by SDS-PAGE and DH%	Best sensory scores: 8.0 ± 0.65 vs. 7.7 ± 0.73 (TCP) in the available study	Limited to one primary study; requires independent replication	Promising potential; scalability not yet independently established	Single study; below 5-log_10_ target; multi-strain and industrial-scale validation absent

**Table 4:** CM packaging technologies in the extension of shelf life: mechanistic reasons, camel-specific science, state of evidence, and priority of research.

Packaging Category	Technology or System	Primary Mechanism	Camel-Specific Scientific Rationale	Evidence Status	Research Priority
**Active: oxygen management**	Oxygen-scavenging films and sachets	Actively absorbs O_2_ from headspace, preventing hydroperoxide initiation cascade	Elevated PUFA content and smaller fat globule size (1.1–2.1 µm) create greater intrinsic oxidative susceptibility than BM; passive barrier packaging insufficient once O_2_ permeates headspace	Commercialized for long-shelf life bovine dairy products; no camel-specific quantitative validation (MDA thresholds or sensory exceedance data)	High
**Active: antimicrobial**	Films incorporating nisin, chitosan, or plant-derived essential oils (spearmint, wild thyme)	Controlled release of antimicrobial agents to product-contact surface or headspace	Functions as a secondary, processing-independent antimicrobial barrier; particularly relevant after HPP or UHT partially degrades intrinsic LF, LZ, and IgG fraction; complements rather than replaces endogenous antimicrobial system	Direct EO addition to CM demonstrated to extend shelf life [11,67]; EO-incorporated film delivery in CM entirely untested	High
**Active: moisture regulation**	Desiccant and humidity buffering films	Maintains optimal water activity to retard Maillard browning and limit microbial proliferation during powder storage	CM powder Maillard kinetics maximized at intermediate a*w* (0.4–0.7); distinct total solid content (~11% vs. ~12% bovine) and mineral composition affect glass transition temperature; moisture control is a direct intervention point	Established for BM powder packaging; CM-specific a*w* thresholds and Maillard rate parameters not validated	Moderate
**Intelligent: time–temperature indicators (TTIs)**	Enzymatic, chemical, or electrochemical Time–temperature indicator labels	Provides cumulative temperature exposure record to detect cold chain abuse before label date	Cold chain failures are disproportionately damaging for CM due to simultaneous acceleration of microbial growth, lipid oxidation, and proteolysis in remote arid supply routes; conventional best-before dating inadequate for highly variable distribution conditions	Dairy context performance documented; CM deterioration rate constants and threshold values for Time–temperature indicator calibration entirely absent	High
**Intelligent: freshness indicators**	pH-responsive color sensors; MDA-reactive or CO_2_-detecting films	Real-time detection of spoilage metabolites without opening the package	CM spoilage volatile profile and MDA accumulation kinetics differ from BM due to compositional differences; sensor calibration must be specific to CM deterioration markers	Dairy context documented; CM-specific calibration entirely absent; requires systematic characterization of spoilage volatile and pH profiles	Moderate
**Bio-based: concept stage**	CM WP isolate films (LF- and LZ-enriched)	Intrinsic antimicrobial activity from LF and LZ embedded in protein film matrix; creates circular economy model within camel dairy industry	Camel whey lacks β-LG, enriched in LF (0.18–2.48 mg/mL) and LZ (228–500 µg/100 mL); antimicrobial activity well-established in aqueous matrices; translation to film architecture scientifically plausible but entirely undemonstrated	No published primary studies; theoretical concept only; fabrication, characterization, and shelf life performance all unvalidated	Future priority
**Bio-based: concept stage**	Chitosan-based antimicrobial films	Polycationic disruption of microbial membranes; biodegradable; compatible with dairy product food-contact regulatory frameworks	Potential synergy with CM’s natural antimicrobial system; cleaner-label alternative to synthetic preservatives; applicable to chilled pasteurized and UHT product formats	Dairy context established; no CM-specific application data	Future priority

*Sources:* [8,11,25,38,42,64,67].

## Data Availability

No new data were created or analyzed in this study.

## Abbreviations

The following abbreviations are used in this manuscript:

AGEs	Advanced glycation end-products
α-LA	α-Lactalbumin
β-LG	β-Lactoglobulin
BM	Bovine milk
CDR3	Complementary determining region 3
CH1	First heavy chain constant region
CM	Camel milk
CN	Casein
CSA	Camel serum albumin
DBD	Dielectric barrier discharge
DH%	Degree of hydrolysis
DPPH	2,2-Diphenyl-1-picrylhydrazyl
ELISA	Enzyme-linked immunosorbent assay
EM	Electromagnetic
FTIR	Fourier-transform infrared spectroscopy
HCAbs	Heavy chain-only antibodies
HMF	Hydroxymethylfurfural
HPP	High-pressure processing
HTST	High-temperature short-time
Ig	Immunoglobulin
IgG	Immunoglobulin G
LF	Lactoferrin
LP	Lactoperoxidase
LTLT	Low-temperature long-time
LZ	Lysozyme
MDA	Malondialdehyde
OPA	o-Phthalaldehyde
PEF	Pulsed electric field
PGRP	Peptidoglycan recognition protein
PUFA	Polyunsaturated fatty acids
RONS	Reactive oxygen and nitrogen species
SANRA	Scale for the Assessment of Narrative Review Articles
SDS-PAGE	Sodium dodecyl-sulphate polyacrylamide gel electrophoresis
TBARS	Thiobarbituric acid reactive substances
TCP	Conventional thermal pasteurization
TTI	Time–temperature indicator
UHT	Ultra-high temperature
UV-C	Ultraviolet C radiation (200–280 nm)
VHH	Variable domain of heavy chain-only antibody (nanobody)
WP	Whey protein
